# Prognostic factors for overall survival in castration-resistant metastatic prostate cancer treated with docetaxel (MeProCSS): results from a German real-world cohort

**DOI:** 10.1007/s11255-025-04389-2

**Published:** 2025-01-27

**Authors:** Felix Steffens, Frederik Wessels, Svetlana Hetjens, Nicolas Carl, Katja Nitschke, Daniel Uysal, Nadim Moharam, Paul Patroi, Thomas Stefan Worst, Karl Friedrich Kowalewski, Maurice Stephan Michel, Manuel Neuberger

**Affiliations:** 1 Department of Urology and Urosurgery, Medical Faculty Mannheim, University Medical Centre Mannheim (UMM), University of Heidelberg, Theodor-Kutzer-Ufer 1-3, 68167 Mannheim, Baden-Württemberg Germany; 2 Department of Medical Statistics and Biomathematics, Medical Faculty Mannheim, University of Heidelberg, Mannheim, Germany

**Keywords:** Inflammatory biomarkers, mGPS, Combined survival score, Docetaxel, Kaplan–Meier estimate

## Abstract

**Purpose:**

To identify prognostic factors for overall survival (OS) and develop a prognostic score in patients receiving docetaxel in metastatic castration-resistant prostate cancer (mCRPC).

**Methods:**

Retrospective analysis was conducted on mCRPC patients treated with docetaxel at a German tertiary center between March 2010 and November 2023. Prognostic clinical and laboratory factors were analyzed using uni- and multivariable logistic regression. Next, the result of the modified Glasgow Prognostic Score (mGPS), neutrophil-to-lymphocyte ratio (NLR) (cut-off ≥3), the presence of high-volume bone metastases (as defined by CHAARTED criteria), hemoglobin (Hb) (cut off < 13.2 g/dl), Gleason score ≥8, and presence of visceral metastases were combined into the Metastasized Prostate Cancer Survival Score (MeProCSS). Patients were then stratified into three prognostic groups. Their OS was assessed by Kaplan–Meier analysis.

**Results:**

Median OS for the overall cohort (*n* = 153) and the first-line cohort (*n* = 83) was 18 and 21.5 months, respectively. In multivariable analysis, high-volume bone metastases and Hb levels below the norm were significant predictors of shorter OS in the total cohort. The MeProCSS demonstrated an area under curve (AUC) of 0.837 in the overall cohort and 0.946 in first-line cohort. Kaplan–Meier analysis revealed a significant association between lower MeProCSS and longer OS in both the overall (*p*<0.001) and first-line (*p* = 0.035) cohort.

**Conclusion:**

MeProCSS, consisting of routinely collected parameters prior to the start of chemotherapy, seems to effectively stratify patients with mCRPC into risk groups based on their metastatic burden, nutritional and inflammatory status. This model may guide treatment decisions and reveal a potentially often underestimated or overlooked urgency for additional measures as supportive palliative care in mCRPC patients. Further large and prospective studies are necessary for validation of MeProCSS—also in other systemic PC therapy regimens.

## Introduction

Prostate cancer (PC) is the second most common malignancy in men worldwide [1]. In 2024, an estimated 299,010 new cases will account for 14.9 % of all cancer diagnoses [2]. By 2040, the incidence of PC is projected to increase by 71.6 % [1].

Once PC metastasizes, it frequently becomes resistant to androgen deprivation therapy (ADT), progressing to metastatic castration-resistant prostate cancer (mCRPC) [3]. Over recent decades, the approval of new therapeutic agents such as docetaxel, abiraterone and olaparib has expanded treatment options for mCRPC. However, selecting the optimal therapeutic agent remains challenging. Several studies have shown the benefits of adding docetaxel to ADT [4, 5]. More recently, various studies have explored the prognostic value of inflammatory markers in patients undergoing treatment with docetaxel and other therapies [6, 7]. These inflammatory markers have been associated with poorer overall survival (OS) in patients with mCRPC. Docetaxel remains recommended for fit patients with mCRPC, as per European Association of Urology (EAU) guidelines [8]. Independent prognostic factors for OS include prior estramustine use, bone scan progression, pain, visceral metastases, and anemia [9].

Even though the therapy landscape and OS of metastatic PC has dramatically improved in the last years and despite the routine assessment of biomarkers, there is still a lack of reliable tools for predicting treatment response and prognosis in patients with mCRPC. Investigations into new biomarkers, tumor genomic sequencing, and cell-free DNA changes have aimed to improve therapeutic decision-making [10]. However, these methods are often expensive and not widely accessible. In contrast, routine clinical practice already includes cost-effective inflammatory markers, making them valuable for day-to-day decision-making.

Therefore, over the years, various individual and combined inflammatory markers were examined. Randomized clinical trials have underscored the value of combined systemic inflammation-based prognostic scores in cancer such as the neutrophil-to-lymphocyte ratio (NLR), systemic immune-inflammation index (SII), and modified Glasgow Prognostic Score (mGPS) [11–13]. In a systematic review of inflammatory markers, most evidence exists for the NLR followed by mGPS [14]. Whereas the NLR only considers two subgroups of white blood cells, the mGPS combines an inflammatory marker (C-reactive protein (CRP)) and a nutritional marker (albumin), two parameters commonly measured in clinical practice. Recent evidence suggested that poor nutrition might be predictive for survival in PC [15]. The mGPS has been linked to clinical outcomes across various cancer types and has recently been associated with OS and disease progression in mCRPC, suggesting it could serve as a useful prognostic tool [7, 16].

The aim of the study was to develop and examine a prognostic score prior to chemotherapy based on real-world data. Further, this study seeks to add more evidence on the suitability of this method for predicting treatment response and OS.

## Materials and methods

### Study population and data collection

**Fig. 1:**
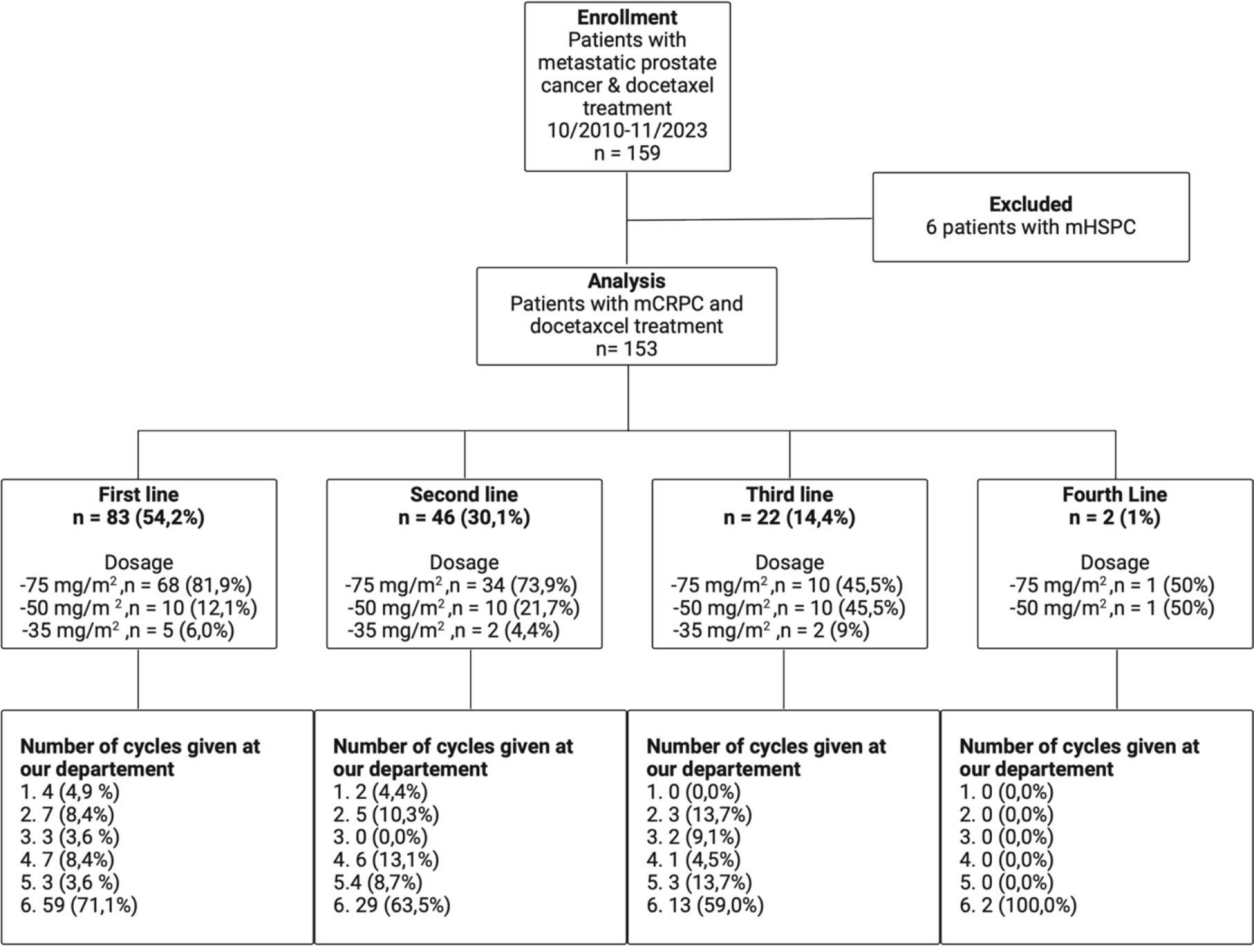
Flow-diagram and docetaxel treatment information of study cohort. *mHSPC* metastatic hormone-sensitive prostate cancer; *mCRPC* metastatic castration-resistant prostate cancer

Patients who received taxane-based chemotherapy and ongoing androgen deprivation treatment at a tertiary university hospital (Mannheim University Hospital, Heidelberg University) in Germany between March 2010 and November 2023 were included. These patients were screened for mCRPC status and docetaxel treatment, as illustrated in Fig. 1. Routine blood test and clinical parameters were assessed prior to each cycle of chemotherapy. Next, in literature research, we identified clinical and laboratory factors that are already used in metastatic PC in various studies and classifications (e.g., for treatment decision) and for which there is statistical evidence as prognostic factors. The most prominent clinical criteria in systemic therapy, especially at the time of initial diagnosis of metastatic prostate cancer are the CHAARTED and LATITUDE criteria, as these can be used to classify high- and low-volume as well as high- and low-risk disease. Both criteria have a certain intersection. To cover these criteria without double weighting the bone metastases, we have opted for high-volume bone metastases as defined in the CHAARTED trial (four or more bone lesions with at least one beyond the vertebral bodies and pelvis [17]), visceral metastases and Gleason ≥8. A growing body of evidence highlights the inflammatory response associated with malignancy [18]. NLR and mGPS were selected because they are the inflammatory markers with the most evidence as prognostic markers [14]. Another reason for these two biomarkers is that they cover complementary inflammatory markers. Therefore, they avoid double entry of neutrophils, which would be present in the systemic inflammatory index, for example. In addition, the mGPS contains information about the nutritional status. For the NLR, the widely acknowledged cut-off of three was used as different studies could show a significant association of poorer prognosis in patients with mCRPC [14]. The mGPS was calculated by definition and as showed in Table 2. Hb was selected since there it is a well-established biomarker that is associated with prognosis in mCRPC [9, 19]. The reference value for anemia in men is given by our laboratory as 13.2 g/dl and, was therefore, used as a cut-off for this analysis. The definitions and results are provided in Table 1, whereas Table 2 shows the components of the MeProCSS for OS analysis. We decided to analyze the laboratory parameters prior to chemotherapy since these cannot be influenced by the body’s reaction to chemotherapy itself. If a specific factor was present, or if the threshold of a laboratory parameter was reached, one point was assigned. The mGPS is an exception since by definition 0, 1 and 2 points can be achieved. Because the mGPS covers the inflammation on the one side and nutritional status on the other side, the mGPS is the only parameter for which 2 points could be awarded. Assigning only one point to the mGPS would have reduced the discriminatory power of this biomarker that has been validated in multiple studies.

The maximum MeProCSS was 7, and based on this, patients were categorized into three prognostic risk groups: a good prognosis group (MeProCSS 1, equals scores 0 to 2), an intermediate prognosis group (MeProCSS 2, equals scores 3 to 4), and a poor prognosis group (MeProCSS 3, equals scores 5 to 7). Since the mGPS is a widely established prognostic score consisting of two components (CRP and albumin), it is the only part of the score for which up to two points are awarded. April 5^th^ 2024 was used to query the death registers, thus marking the end date of the survival analysis. Clinical and demographic data were obtained from the patients’ medical records.

## Ethics approval

This study was conducted according to the Declaration of Helsinki; all patients gave their written informed consent. This study was approved by the local ethics committee (University of Heidelberg’s Ethics Committee II, Medical Faculty Mannheim, reference number 2023-845-AF 11).

## Statistical analyses

Descriptive features were used for cohort characterization: medians and inter-quartile ranges (IQR) were computed for continuous variables and frequencies and proportions were established for categorical variables. Using the Cochran-Armitage Trend Test, the inflammatory markers were evaluated for their potential as survival predictors. Chi-squared test was used to examine the significance of the association between categorical variables and overall survival. Moreover, the influence of clinical and laboratory factors on the OS were examined using uni- and multivariable logistic regression models.

Independent prognostic markers were identified using multivariable logistic regression. Log rank testing and Kaplan–Meier analyses were used for the survival analyses. All tests were two-sided. The threshold for statistical significance was set at α = 0.05. The calculations were performed using SAS^®^ 9.4 (SAS Institute Inc., Cary, North Carolina, USA) and JMP^®^ *16.0.0* (SAS Institute Inc., Cary, NC, 1989–2023). Illustrations were created with BioRender.com. RStudio was used for Time-to-Event analysis (Version 2024.09.0+375, Packages used: ggplot2, survminer &survival). The statistical code was trivial but can be provided upon reasonable request from the corresponding author.

## Results

Eighty-three patients (54.2%) received docetaxel as their first line of treatment. Table 1 presents a comprehensive clinical cohort characterization. The median OS for the first-line cohort was 21.5 (IQR 12–47.8) months, while the median OS for the entire cohort was 18 (IQR 8–32) months. A detailed clinical characterization of the cohort is provided in Fig. 1 and Table 1.

**Table 1:** Baseline characteristics of study cohort

Characteristic	Total cohort (*n* = 153)	First line cohort (*n* = 83)
Age [years], median [IQR]	72 [65–76]	71 [63–76]
Gleason score ≥ 8 (*n*, %)	94 (71.2 )^a^	52 (72.2)^n^
PSA [ng/ml] at 1st cycle, median [IQR]	63.3 [15,21–257.83]^b^	50.8 [9.4–220.8]^o^
Lymphatic metastases (*n*, %)	88 (57.5)	49 (59.0)
Bone metastases (*n*, %)	129 (84.3)	62 (74.7)
Hepatic metastases (*n*, %)	23 (15)	14 (16.9)
Visceral metastases (*n*, %)	32 (20.9)	18 (21.7)
Hb [g/dl], median [IQR]	12.35 [10.3–13.4]^c^	12.6 [10.4–13.6]
NLR, median [IQR]	4.1 [2.8–6.2]^d^	4.0 [2.5–6.3]^n^
Albumin [g/dl], median [IQR]	35.8 [31.8–39.0]^e^	36.9 [32.9–39.9]
CRP > 10 [mg/l] (*n*, %)	65 (44.2)^f^	36 (45.6)^p^
mGPS^g,q^		
− 0 (*n*, %)	76 (53.5)	37 (48.6)
− 1 (*n*, %)	23 (16.2)	22 (28.9)
− 2 (*n*, %)	43 (30.3)	17 (22.5)
MeProCSS^h, r^		
− 0 (*n*, %)	2 (2)	2 (6)
− 1 (*n*, %)	11 (10)	6 (15)
− 2 (*n*, %)	19 (18)	11 (22)
− 3 (*n*, %)	27 (25)	12 (22)
− 4 (*n*, %)	19 (18)	12 (20)
− 5 (*n*, %)	15 (14)	8 (11)
− 6 (*n*, %)	12 (11)	3 (4)
− 7 (*n*, %)	3 (2)	0 (0)
High volume (*n*, %) (CHAARTED)	88 (60.7)^i^	43 (55.8)^s^
High Risk (*n*, %) (LATITUDE)	75 (56.4)^j^	37 (52.1)^t^
De Novo metastases (*n*, %)	85 (55.9)^k^	48 (58,5)^u^
Metachronous metastasis (*n*, %)	67 (44.1)^l^	34 (41,5)^v^
Overall Survival [months], median [IQR]	18 [8–32]^m^	21.5 [12–47.8]^w^

*IQR* interquartile range, PSA prostate-specific antigen, *Hb* hemoglobin, *NLR* neutrophil-to-lymphocyte ratio. *CRP* C-reactive protein, *mGPS* modified Glasgow prognostic score, *MeProCSS* metastasized prostate cancer survival score

^a^Data of 21 patients missing

^b^Data of 2 patients missing

^c^Data of 1 patient missing

^d^Data of 14 patients missing

^e^Data of 7 patients missing

^f^Data of 6 patients missing

^g^Data of 11 patients missing in total cohort

^h^Data of 45 patients missing

^i^Data of 8 patients missing

^j^Data of 20 patients missing

^k^Data of 1 patient missing

^l^Data of 1 patient missing

^m^Data of 16 patients missing

^n^Data of 11 patients missing

^o^Data of 1 patient missing

^p^Data of 4 patients missing in first-line cohort

^q^Data of 7 patients missing

^r^Data of 29 patients missing

^s^Data of 6 patients missing

^t^Data of 12 patients missing

^u^Data of 1 patient missing

^v^Data of 1 patient missing

^w^Data of 7 patients missing

**Table 2:** Components of metastasized prostate cancer survival score (MeProCSS)

mGPS	
C-reactive protein ≤ 10 mg/l and any albumin value	0
C-reactive protein > 10 mg/l and albumin ≥ 35 g/l	1
C-reactive protein > 10 mg/l and albumin < 35 g/l	2
NLR > 3	1
neutrophile count (reference range∶ 4−10×109∕L)	
lymphocyte count (reference range∶ 1.1−3.2×10 ∕L)	
Hb [g/dl] ≤ 13.2	1
Gleason score ≥ 8 (ISUP ≥ 4)	1
Visceral metastases	1
High-volume bone metastases (CHAARTED criteria)	1

*mGPS* modified Glasgow prognostic score, *NLR* neutrophil-to-lymphocyte ratio, *Hb* hemoglobin, *ISUP* International Society of Urologic Pathologists

## Total cohort

**Fig. 2:**
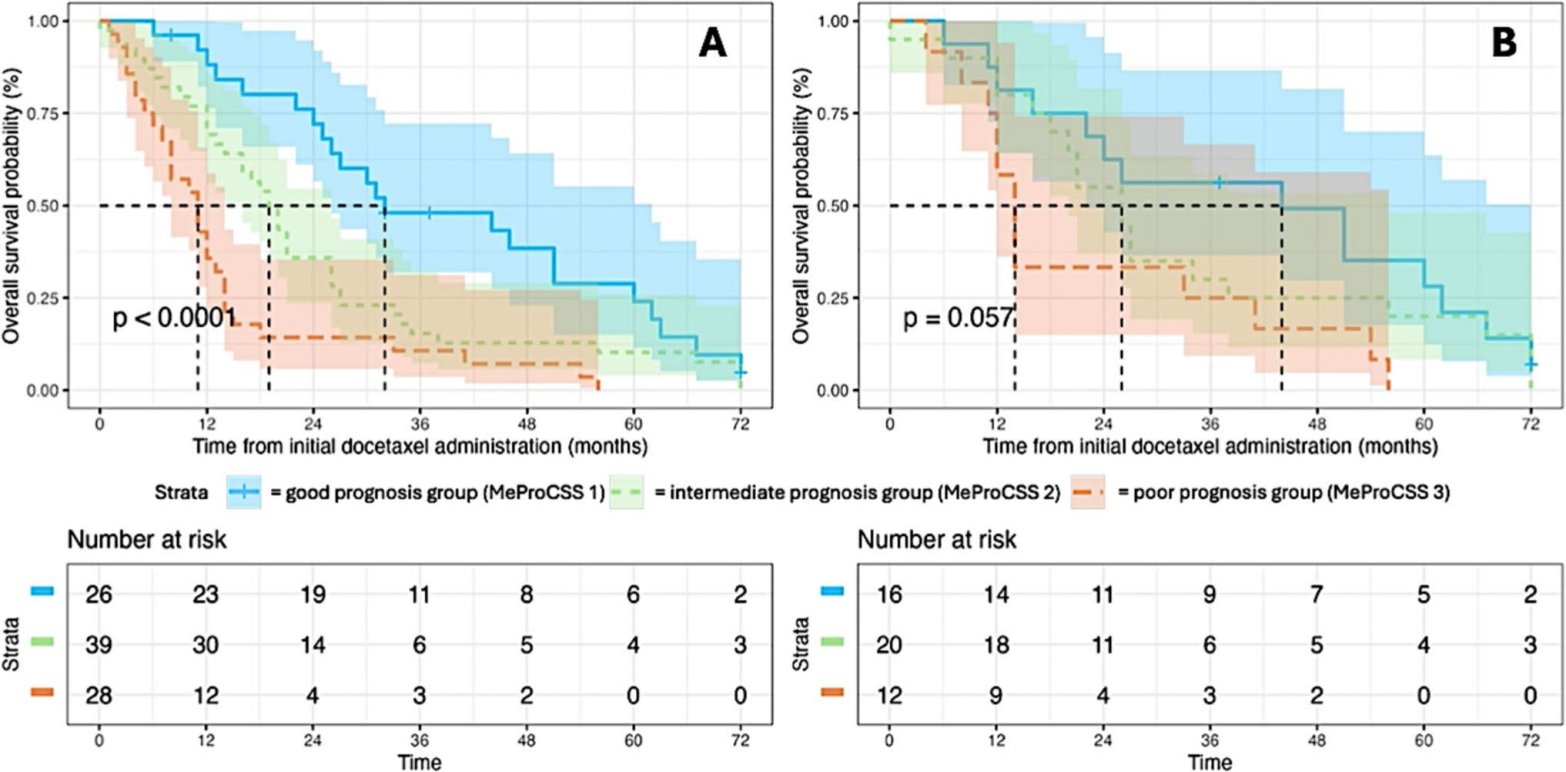
Kaplan–Meier analysis of overall survival (OS) [months] depending on **A** metastasized prostate cancer survival score (MeProCSS) in the total cohort **B** MeProCSS in the docetaxel first-line subgroup

In the total cohort, univariable logistic regression identified mGPS (OR 0.326, 95% CI 0.12–0.886, *p* = 0.020) and Hb (OR 0.107, 95% CI 0.028–0.406, *p* = 0.001) as statistically significant factors associated with OS. High-volume bone metastases (OR 6.844, 95% CI 1.199–39.062, *p* = 0.03) and Hb levels below the reference value of 13.2 g/dl (OR 5.22, 95% CI 01.116–24.406, *p* = 0.035) remained independent predictors of worse OS in multivariable logistic regression analysis. The results are presented in Table 3. MGPS, NLR, Gleason score ≥ 8 and visceral metastases showed no significant association in multivariable logistic regression analysis. The area under curve (AUC) for MeProCSS in the total cohort was 0.837, referring to the predictive power in multivariable logistic regression analysis. Kaplan–Meier analysis revealed that patients with lower MeProCSS scores had significantly longer OS compared to those with higher MeProCSS scores (median survival: MeProCSS 1 (0–2 points) = 32 months, MeProCSS 2 (3–4 points) = 19 months, MeProCSS 3 (5–7 points) = 11 months, *p* < 0.001). These results are presented in Fig. 2.

## First-line cohort

In the first-line cohort, no significant association was found between the six evaluated markers and OS in either univariable or multivariable logistic regression analyses. The AUC for MeProCSS in the first-line cohort was 0.946, referring to the predictive performance in multivariable logistic regression. The results are summarized in Table 3. Kaplan–Meier analysis demonstrated that patients with lower MeProCSS scores had longer OS compared to those with higher scores (median survival: MeProCSS 1 (0–2 points) = 44 months, MeProCSS 2 (3–4 points) = 26 months, MeProCSS 3 (5–7 points) = 12 months, *p* < 0.035). These findings are shown in Fig. 2.

**Table 3:** Uni- and multivariable logistic regression for overall survival

Total cohort (*n* = 153 )						
	Univariable analysis			Multivariable analysis		
	OR	95% CI	p	OR	95% CI	p
mGPS (per unit)	0.326	0.120–0.886	**0.020**	0.151	0.019–1.192	0.072
HB (< 13.2 mg/dl)	0.107	0.028–0.406	**0.001**	5.22	1.116–24.406	**0.035**
NLR (over 3)	1.481	0.463–4.745	0.508	0.513	0.107–2.449	0.402
Gleason Score ≥ 8 (yes vs. no)	1.416	0.440–4.560	0.559	1.447	0.325–6.44	0.627
Visceral disease (yes vs. no)	1.784	0.380–8.376	0.463	1.283	0.127–12.942	0.832
High-volume bone metastases (CHAARTED criteria)	3.735	0.980–14.232	0.053	6.844	1.199–39.062	**0.030**

Firstline cohort (n = 83)						
	Univariable analysis			Multivariable analysis		
	OR	95% CI	p	OR	95% CI	p
mGPS (per unit)	0.535	0.170–1.68	0.284	0.080	0.003–2.561	0.154
HB (under 13.2 mg/dl)	<0.001	<0.010–>999.99	0.949	>999.99	<0.001–>999.999	0.896
NLR (over 3)	1.048	0.170–6.180	0.959	0.110	0.005–2.331	0.157
Gleason Score ≥ 8 (yes vs. no)	1.941	0.390–9.590	0.416	14.784	0.594–368.236	0.100
Visceral disease (yes vs. no)	1.714	0.190–15.97	0.629	>999.999	<0.001–> 999.999	0.959
High-volume bone metastases (CHAARTED criteria) (yes vs. no)	1.892	0.340–10.470	0.465	11.051	0.290–421.215	0.195

Bold values indicate statistically significant differences (*p* < 0.05)

*mGPS* modified Glasgow prognostic score, *NLR* neutrophil-to-lymphocyte ratio, *Hb* hemoglobin, *CI* confidence interval, *OR* odds ratio

## Discussion

Recent advances in the treatment of mCRPC have introduced new therapeutic alternatives, highlighting the growing need for individualized and optimized treatment selection. Studies such as CHAARTED and LATITUDE have provided valuable definitions of mCRPC to assess a patient’s disease status [17, 20]. However, these scores primarily consider tumor characteristics, omitting the overall condition of the patient. Despite the significant progress made in treatment options, there are currently no widely implemented prognostic scores for OS in metastatic PC. This study aims to explore and develop a straightforward combined prognostic score based on routine data and that reflect the body’s inflammatory, nutritional, and metastatic status. Such a score could improve patient stratification and assist clinicians in making treatment decisions.

Whereas in univariable analysis Hb and mGPS showed significant association with OS in the total cohort, Hb levels and high-volume bone metastases remained as the only independent predictors of OS in multivariable analysis for the total cohort. However, in the first-line cohort, no significant association was found in either uni- or multivariable analysis. Therefore, the significant results in the multivariable analysis for total cohort are particularly promising. The lack of significant associations in the first-line cohort may be due to the small sample size, as other studies have demonstrated significant associations between variables like Gleason score and survival [16, 21]. As visceral and bone metastases can reflect the biological aggressiveness of the tumor and the extent of metastatic spread, their integration into predictive models seem reasonable and useful.

The elevated AUC values suggests a strong predictive probability for the MeProCSS in relation to OS. The aforementioned lack of significant correlations between single parameters and OS further supports the use of the MeProCSS, as it enables stratification by incorporating multiple clinical and inflammatory markers. A lower MeProCSS score was significantly associated with longer OS in both the total and first-line cohort. These findings support the integration of clinical parameters alongside inflammatory biomarkers for predicting survival in mCRPC.

As a hallmark of cancer and cancer progression [22] and due to its easy assessment through routine blood tests recent research on inflammatory biomarkers in PC has provided insights in their prognostic value in mCRPC [7, 23]. Despite these findings, current PC guidelines (e.g., German S3, EAU, ESMO, AUA) do not yet incorporate these markers. Furthermore, there is a lack of published data regarding the use of these markers before chemotherapy administration. In 2020, Donate-Moreno et al. prospectively examined inflammatory markers in 80 patients with mCRPC undergoing various treatment therapies, demonstrating a negative correlation between NLR, systemic immune-inflammation index (SII), platelet-to-lymphocyte ratio (PLR), and survival [6]. Furthermore, a low pre-treatment NLR has been associated with longer OS in patients with mCRPC receiving first-line docetaxel therapy [24]. Recent studies have emphasized the value of inflammatory markers such as NLR, mGPS, and SII, as prognostic indicators in patients with mCRPC treated with docetaxel or abiraterone after docetaxel therapy [16, 25].

Next to inflammatory markers, the nutritional status reflects the patients clinical condition and their capacity to endure antitumoral therapies [26]. Hypoalbuminemia in malnourished patients has been linked to an elevated risk of chemotherapy-induced toxicity and diminished treatment efficacy, likely due to decreased plasma protein binding of therapeutic agents [26]. Independent of chemotherapy status, recent studies have also shown that the combination of body mass index and albumin predicts OS in mCRPC patients treated with abiraterone [27]. Additionally, quality of life tends to decline in patients with mCRPC and poor nutritional status [28]. Sohlberg et al. demonstrated an association between low albumin and shorter OS in mCRPC [29].

In contrast to other markers, the mGPS combines data from two different sections: the inflammatory and the nutritional status. Various studies have shown a correlation between the mGPS and OS in chemotherapy-naive mCRPC [30], as well as in castration-resistant PC [13], with worse 5-year-survival outcomes independent of age [31]. Furthermore, in mCRPC mGPS has also been linked to progression-free survival [7]. A recently published retrospective study involving 79 patients with mCRPC treated with enzalutamide or abiraterone supported the role of mGPS as a marker for OS [32]. Next to mCRPC, a correlation between OS and mGPS has been observed in patients with metastatic hormone-sensitive prostate cancer (mHSPC), further supporting the utility in predicting patient outcomes [33].

Apart from the mGPS there have been retrospective studies to evaluate the added value of combined information: Smaletz et al. aimed to generate nomograms based on pretreatment clinical parameters for predicting OS in mCRPC, combining factors such as Karnofsky performance status (KPS), Hb, PSA, lactate dehydrogenase (LDH), alkaline phosphatase (ALK) and albumin [34]. This nomogram, however, provided only moderate accuracy and did not include inflammatory or nutritional biomarkers. Ando et al. developed a prognostic score incorporating high-sensitivity mGPS, PSA and serum testosterone levels to predict outcomes in patients with mCRPC undergoing docetaxel therapy [13]. A significant association with OS was observed, supporting the prognostic value of inflammatory and nutritional markers. Yazgan et al. demonstrated a significant correlation between OS and a combined score based on neutrophil, platelet, monocyte, and lymphocyte counts [35]. Notably, nutritional status was not included. To date, none of the approaches mentioned has been widely adopted by professionals in clinical practice.

In other urological malignancies like urothelial carcinoma, kidney cancer, and penile cancer, the prognostic value of mGPS as a simple “combined” score based on two biomarkers has been well documented [36–39]. Additionally, a recent study demonstrated the prognostic significance of on-treatment mGPS for OS in metastatic renal cell carcinoma treated with atezolizumab plus bevacizumab or sunitinib [40]. To date, no studies have successfully established a combined score system for urological malignancies that integrated inflammatory, nutritional, and clinical parameters to provide reliable predictive insights. In metastatic kidney cancer, the International Metastatic Renal Cell Carcinoma Database Consortium (IMDC) score [41] serves as a meaningful example, incorporating various laboratory and clinical parameters to inform drug approval and treatment decisions.

Systemic inflammatory biomarkers have been evaluated in a multitude of malignancies. To name only a few, a recent meta-analysis by Jarmuzek et al. (2023) demonstrated significantly worse outcomes for patients with glioblastoma and high NLR and PLR [42]. Similarly, Lorton et al. reported a significant association between elevated mGPS and reduced OS in esophageal cancer [43]. A Danish register-based study showed a significant correlation between OS and a combined biomarker score including Hb, albumin, CRP, neutrophil and lymphocyte count in patients with non-small cell lung cancer [44]

The decision to pursue double or triple systemic therapy in mHSPC is largely influenced by high- or low-volume status, as defined by the CHAARTED criteria [17, 45]. Therefore, markers such as Gleason score, as well as visceral or high-volume bone metastases appear to be promising since reflecting the biological aggressiveness of the tumor and the extent of metastatic spread.

The main limitation is the study’s statistical power and generalizability due to the relatively small sample size and missing data, which led to the exclusion of some patients for the analysis of the MeProCSS. 64 of 153 patients of the total and 38 of 83 patients of the first-line cohort were excluded for calculation of the MeProCSS due to missing variables. Thus, the first line-cohort is rather small and only 12 patients were attributed to the poor prognosis group. The small group size might bias our results and shows that - even though the AUC values are promising and indicate potential predictive potential—our results, but especially the subgroup results for the first-line cohort should be interpreted cautiously.

Moreover, the retrospective nature and single-center design of this study limits external validity. Additional limitations are missing comorbidities and cause of death and the heterogenous study population of this real-world cohort with docetaxel in different therapy lines. However, these limitations and the inclusion of docetaxel in first-, second-, third- or fourth-line therapy in mCRPC, could be cautiously interpreted to mean that the MeProCSS has a certain generalizability.

The significant association of the MeProCSS with OS underscores its potential for practical application in real-world clinical settings, although cancer-specific survival could not be calculated in this study. Despite these limitations, the study's real-world setting could enhance the generalizability and robustness of its conclusions, contributing valuable insights into clinical practice. This would help to establish the robustness across different clinical settings. Additionally, comprehensive data from these studies could enhance our understanding of the score’s prognostic and predictive capabilities. These investigations may uncover potential refinements that could improve its accuracy and applicability in routine clinical practice.

The MeProCSS could help clinicians identify patients at higher risk of poor outcomes in various time points of their treatment course. For patients presenting with first diagnosis of mHSPC the MeProCSS could be used alongside the CHAARTED and LATTITUDE criteria [17, 20] and synchronous or metachronous metastasis [46]. Especially in cases with low-volume disease the MeProCSS could help identify patients with poor prognosis and guide treatment decision towards triple therapy. On the other side, especially high-volume patients that present with a higher ECOG or a more vulnerable state could be stratified in the three MeProCSS risk groups to better discuss the possible benefits and harms of an early treatment intensification in terms of a triple therapy. For patients with poor prognosis, an early genomic testing could be helpful, since they might also suffer from a more aggressive disease (e.g., with genetic mutations) and benefit from a consecutive combination therapy in mCRPC (e.g., new hormonal therapy (NHT) + PARP inhibitor (PARPi). On the other hand, the MeProCSS, especially in combination with the ECOG, could be a useful tool to objectively identify patients with poor prognosis in later therapy lines. In times of multiple therapeutic options and remaining uncertainty about the best individual therapy sequence, this might help to discuss either a more intense therapy in case of progression or to help articulate the limited prognosis to also discuss best supportive care instead of further therapies. The MeProCSS could also be used for regular reassessment of the necessity of supportive palliative care whenever systemic therapy is changed. This might be especially interesting before starting mCRPC treatment or later line systemic therapy, because the urgency of palliative involvement, e.g. (specialized) outpatient palliative care, and advance healthcare directive could be revealed/objectified. As the MeProCSS already incorporates the Gleason score, an established risk stratification tool, further studies are necessary to assess its performance and explore its potential integration with other prognostic systems, such as the TNM classification.

Larger and prospective multi-center studies in different therapy regimens are necessary to further evaluate the prognostic and predictive value of the MeProCSS in both mHSPC and mCRPC for broader validation. Considering mHSPC patient cohorts who receive triple therapy (for example with ADT, docetaxel and darolutamide or abiraterone) or testing in mCRPC studies that analyzed PSMA-targeted radioligand therapy or PARPi could be promising. Additionally, MeProCSS could also be tested as a marker for response to systemic therapies in mCRPC setting.

In conclusion, the MeProCSS consists of cost-effective and easily accessible information and biomarkers, that are routinely measured before chemotherapy as well as the new hormonal therapies. It seems to allow for stratification based on metastatic, nutritional, and inflammatory status, and may assist clinicians in treatment decision-making. Furthermore, it may aid in assessing overall prognosis, emphasizing the need for timely and proactive management of both clinical and socio-organizational matters, such as advance healthcare directives.

## Conclusion

In this study, we assessed the potential of MeProCSS as a prognostic score in patients with mCRPC undergoing docetaxel therapy. Baseline MeProCSS appears to be an independent prognostic score for patients with mCRPC receiving docetaxel in routine clinical practice. However, these findings, derived from a retrospective analysis, require confirmation through larger, prospective, multicenter studies.

## Data Availability

The dataset generated during the current study is available from the corresponding author upon reasonable request.
